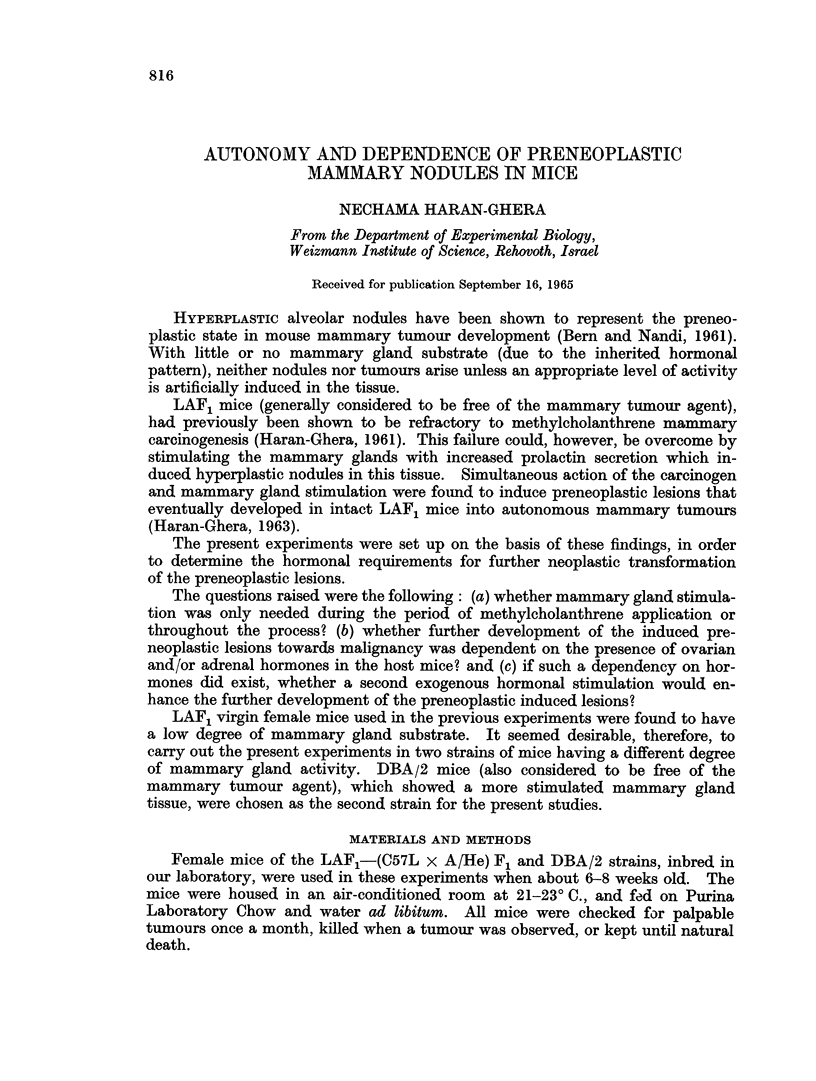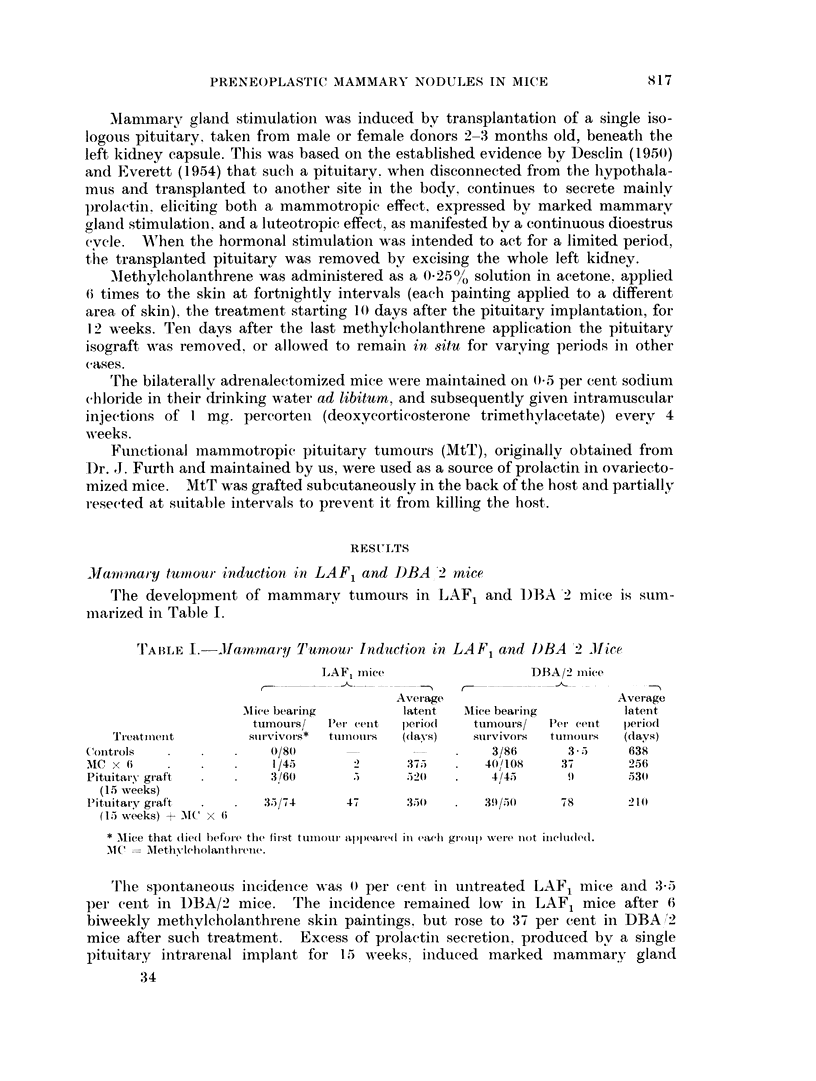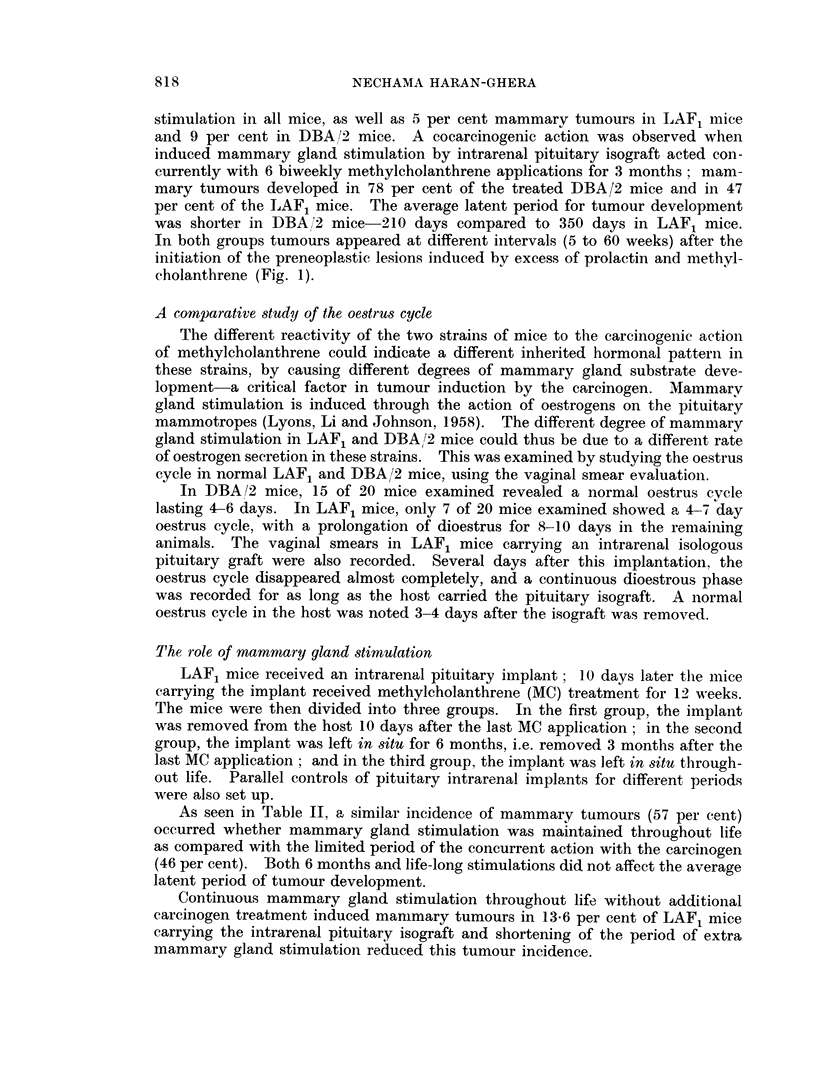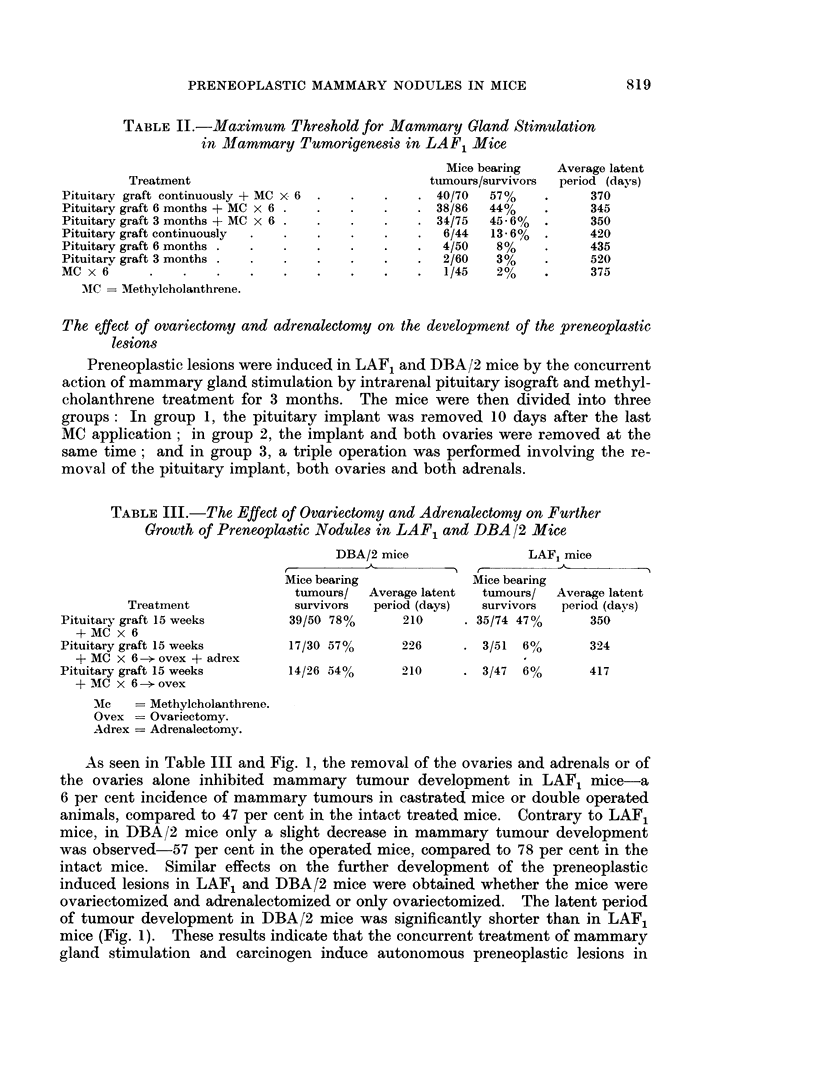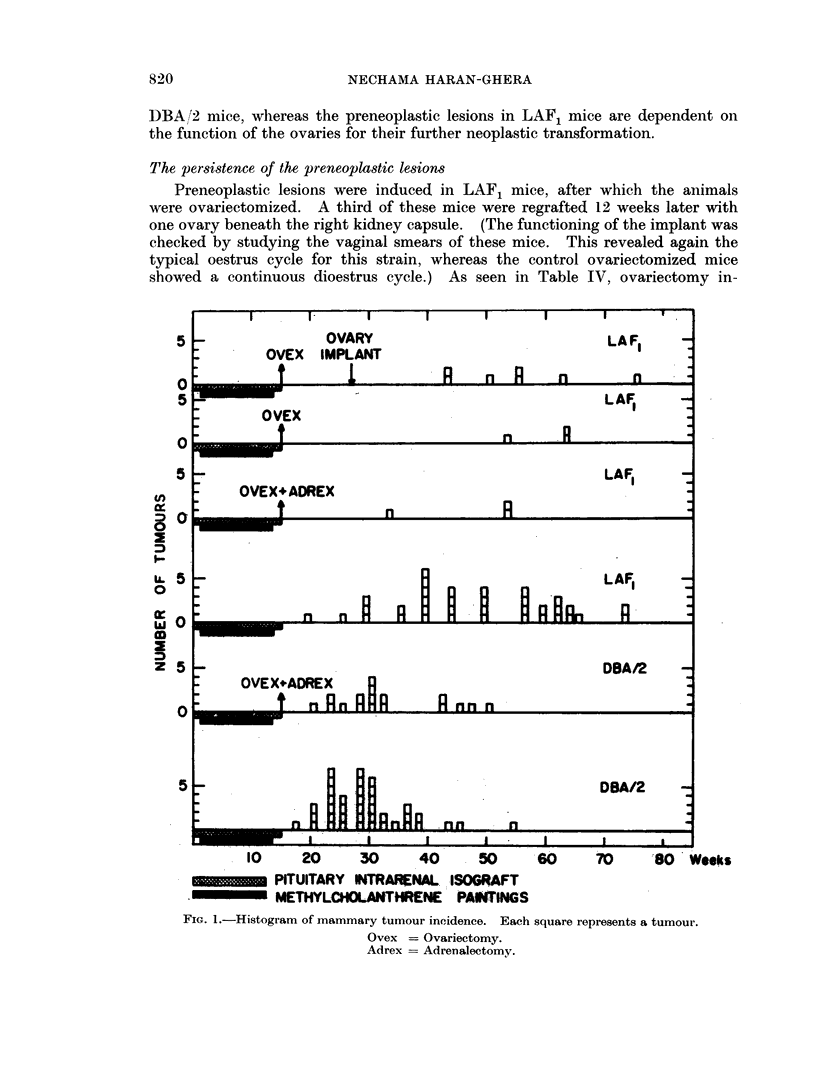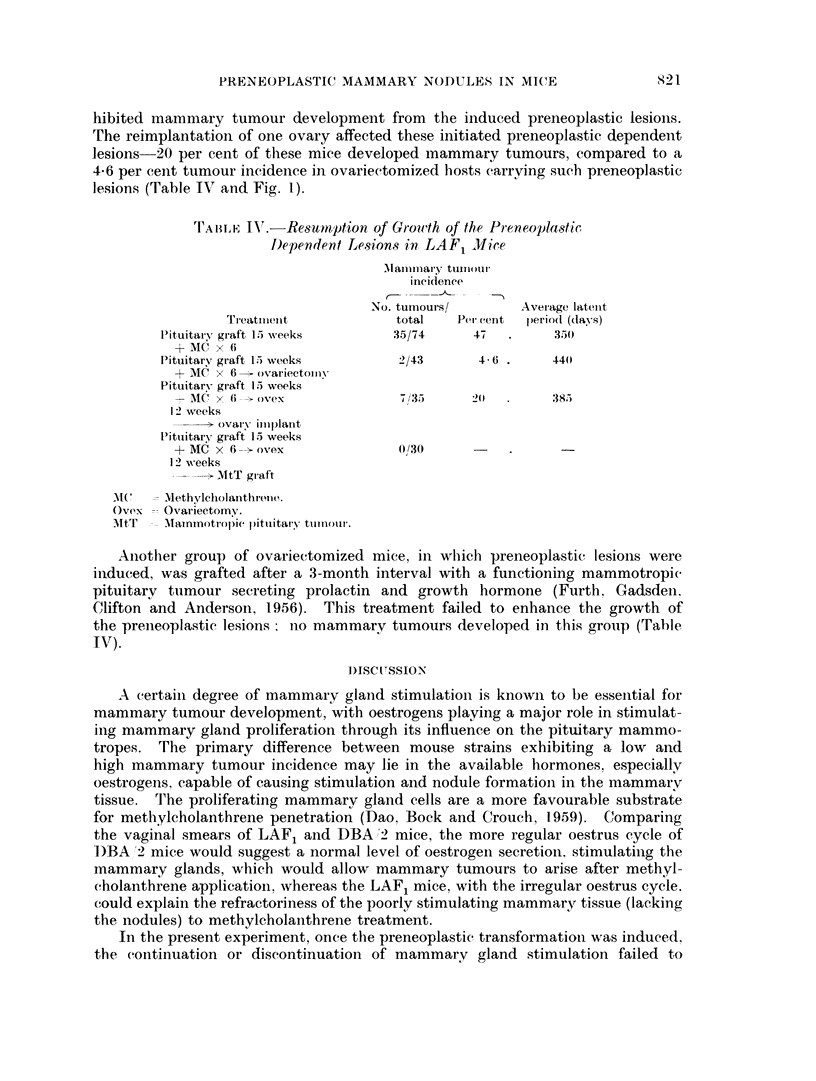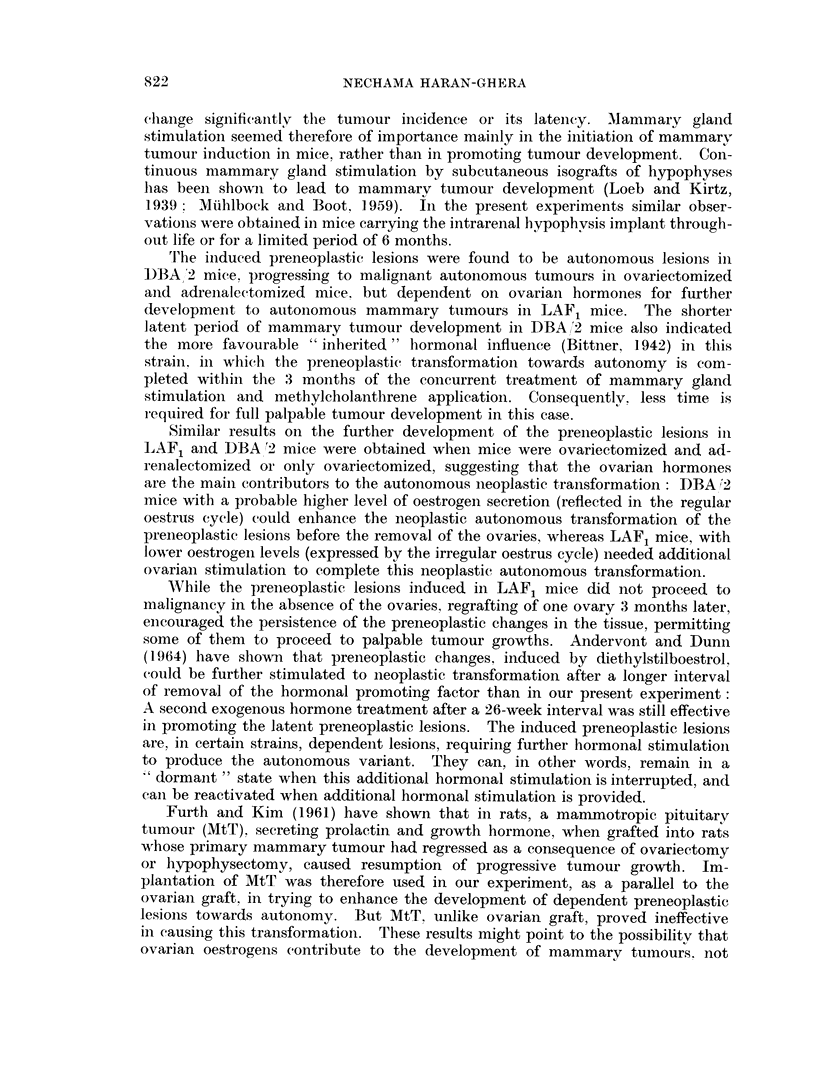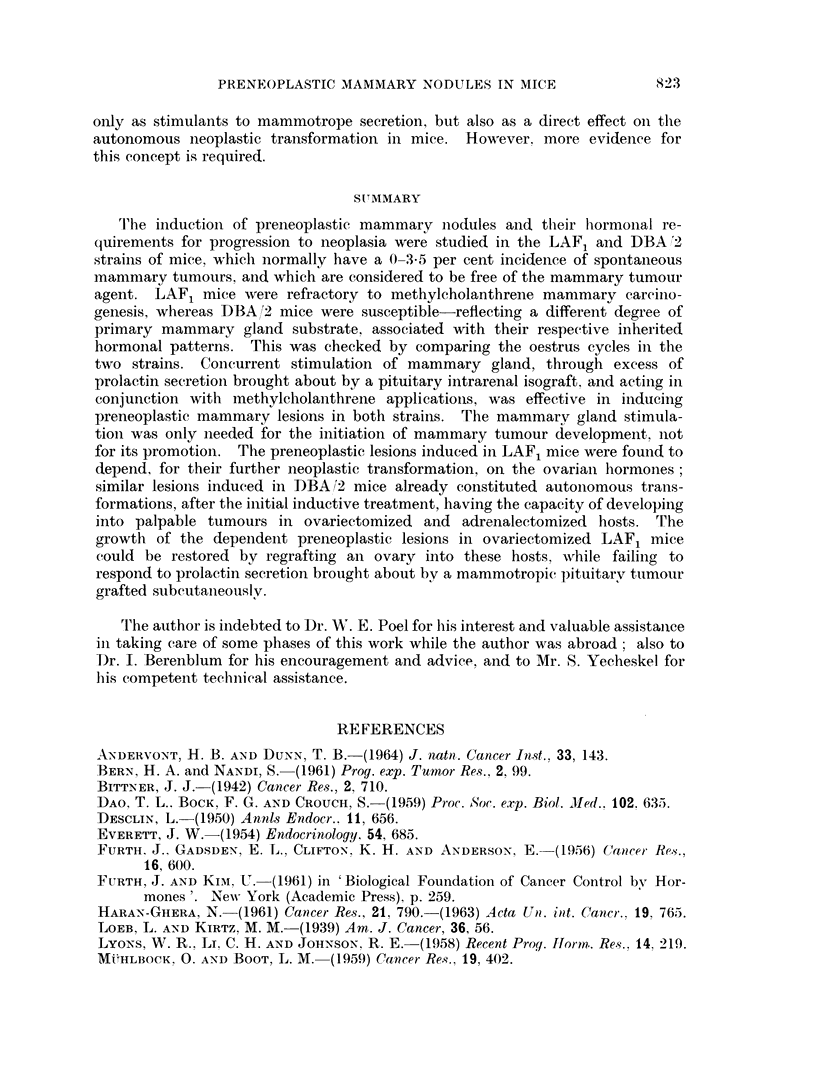# Autonomy and dependence of preneoplastic mammary nodules in mice.

**DOI:** 10.1038/bjc.1965.94

**Published:** 1965-12

**Authors:** N. Haran-Ghera


### AUTONOMY AND DEPENDENCE OF PRENEOPLASTIC MAMMARY NODULES IN MICE

NECHAMA HARAN-GHERA

*From the Department of Experimental Biology, Weizmann Institute of Science, Rehovoth, Israel*

Received for publication September 16, 1965

HYPERPLASTIC alveolar nodules have been shown to represent the preneoplastic state in mouse mammary tumour development (Bern and Nandi, 1961). With little or no mammary gland substrate (due to the inherited hormonal pattern), neither nodules nor tumours arise unless an appropriate level of activity is artificially induced in the tissue.

$LAF_1$ mice (generally considered to be free of the mammary tumour agent), had previously been shown to be refractory to methylcholanthrene mammary carcinogenesis (Haran-Ghera, 1961). This failure could, however, be overcome by stimulating the mammary glands with increased prolactin secretion which induced hyperplastic nodules in this tissue. Simultaneous action of the carcinogen and mammary gland stimulation were found to induce preneoplastic lesions that eventually developed in intact $LAF_1$ mice into autonomous mammary tumours (Haran-Ghera, 1963).

The present experiments were set up on the basis of these findings, in order to determine the hormonal requirements for further neoplastic transformation of the preneoplastic lesions.

The questions raised were the following : (*a*) whether mammary gland stimulation was only needed during the period of methylcholanthrene application or throughout the process? (*b*) whether further development of the induced preneoplastic lesions towards malignancy was dependent on the presence of ovarian and/or adrenal hormones in the host mice? and (*c*) if such a dependency on hormones did exist, whether a second exogenous hormonal stimulation would enhance the further development of the preneoplastic induced lesions?

$LAF_1$ virgin female mice used in the previous experiments were found to have a low degree of mammary gland substrate. It seemed desirable, therefore, to carry out the present experiments in two strains of mice having a different degree of mammary gland activity. DBA/2 mice (also considered to be free of the mammary tumour agent), which showed a more stimulated mammary gland tissue, were chosen as the second strain for the present studies.

### MATERIALS AND METHODS

Female mice of the $LAF_1$—(C57L $\times$ A/He) $F_1$ and DBA/2 strains, inbred in our laboratory, were used in these experiments when about 6–8 weeks old. The mice were housed in an air-conditioned room at 21–23° C., and fed on Purina Laboratory Chow and water *ad libitum*. All mice were checked for palpable tumours once a month, killed when a tumour was observed, or kept until natural death.

Mammary gland stimulation was induced by transplantation of a single isologous pituitary, taken from male or female donors 2–3 months old, beneath the left kidney capsule. This was based on the established evidence by Desclin (1950) and Everett (1954) that such a pituitary, when disconnected from the hypothalamus and transplanted to another site in the body, continues to secrete mainly prolactin, eliciting both a mammotropic effect, expressed by marked mammary gland stimulation, and a luteotropic effect, as manifested by a continuous dioestrus cycle. When the hormonal stimulation was intended to act for a limited period, the transplanted pituitary was removed by excising the whole left kidney.

Methylcholanthrene was administered as a 0·25% solution in acetone, applied 6 times to the skin at fortnightly intervals (each painting applied to a different area of skin), the treatment starting 10 days after the pituitary implantation, for 12 weeks. Ten days after the last methylcholanthrene application the pituitary isograft was removed, or allowed to remain *in situ* for varying periods in other cases.

The bilaterally adrenalectomized mice were maintained on 0·5 per cent sodium chloride in their drinking water *ad libitum*, and subsequently given intramuscular injections of 1 mg. percorten (deoxycorticosterone trimethylacetate) every 4 weeks.

Functional mammotropic pituitary tumours (MtT), originally obtained from Dr. J. Furth and maintained by us, were used as a source of prolactin in ovariectomized mice. MtT was grafted subcutaneously in the back of the host and partially resected at suitable intervals to prevent it from killing the host.

### RESULTS

### *Mammary tumour induction in $LAF_1$ and DBA/2 mice*

The development of mammary tumours in $LAF_1$ and DBA/2 mice is summarized in Table I.

TABLE I.—*Mammary Tumour Induction in $LAF_1$ and DBA/2 Mice*

| | $LAF_1$ mice | | | DBA/2 mice | | |
|---|---|---|---|---|---|---|
| Treatment | Mice bearing tumours/ survivors* | Per cent tumours | Average latent period (days) | Mice bearing tumours/ survivors | Per cent tumours | Average latent period (days) |
| Controls | 0/80 | — | — | 3/86 | 3·5 | 638 |
| MC × 6 | 1/45 | 2 | 375 | 40/108 | 37 | 256 |
| Pituitary graft (15 weeks) | 3/60 | 5 | 520 | 4/45 | 9 | 530 |
| Pituitary graft (15 weeks) + MC × 6 | 35/74 | 47 | 350 | 39/50 | 78 | 210 |

* Mice that died before the first tumour appeared in each group were not included.

MC = Methylcholanthrene.

The spontaneous incidence was 0 per cent in untreated $LAF_1$ mice and 3·5 per cent in DBA/2 mice. The incidence remained low in $LAF_1$ mice after 6 biweekly methylcholanthrene skin paintings, but rose to 37 per cent in DBA/2 mice after such treatment. Excess of prolactin secretion, produced by a single pituitary intrarenal implant for 15 weeks, induced marked mammary gland stimulation in all mice, as well as 5 per cent mammary tumours in $LAF_1$ mice and 9 per cent in DBA/2 mice. A cocarcinogenic action was observed when induced mammary gland stimulation by intrarenal pituitary isograft acted concurrently with 6 biweekly methylcholanthrene applications for 3 months ; mammary tumours developed in 78 per cent of the treated DBA/2 mice and in 47 per cent of the $LAF_1$ mice. The average latent period for tumour development was shorter in DBA/2 mice—210 days compared to 350 days in $LAF_1$ mice. In both groups tumours appeared at different intervals (5 to 60 weeks) after the initiation of the preneoplastic lesions induced by excess of prolactin and methylcholanthrene (Fig. 1).

*A comparative study of the oestrus cycle*

The different reactivity of the two strains of mice to the carcinogenic action of methylcholanthrene could indicate a different inherited hormonal pattern in these strains, by causing different degrees of mammary gland substrate development—a critical factor in tumour induction by the carcinogen. Mammary gland stimulation is induced through the action of oestrogens on the pituitary mammotropes (Lyons, Li and Johnson, 1958). The different degree of mammary gland stimulation in $LAF_1$ and DBA/2 mice could thus be due to a different rate of oestrogen secretion in these strains. This was examined by studying the oestrus cycle in normal $LAF_1$ and DBA/2 mice, using the vaginal smear evaluation.

In DBA/2 mice, 15 of 20 mice examined revealed a normal oestrus cycle lasting 4–6 days. In $LAF_1$ mice, only 7 of 20 mice examined showed a 4–7 day oestrus cycle, with a prolongation of dioestrus for 8–10 days in the remaining animals. The vaginal smears in $LAF_1$ mice carrying an intrarenal isologous pituitary graft were also recorded. Several days after this implantation, the oestrus cycle disappeared almost completely, and a continuous dioestrous phase was recorded for as long as the host carried the pituitary isograft. A normal oestrus cycle in the host was noted 3–4 days after the isograft was removed.

*The role of mammary gland stimulation*

$LAF_1$ mice received an intrarenal pituitary implant ; 10 days later the mice carrying the implant received methylcholanthrene (MC) treatment for 12 weeks. The mice were then divided into three groups. In the first group, the implant was removed from the host 10 days after the last MC application ; in the second group, the implant was left *in situ* for 6 months, i.e. removed 3 months after the last MC application ; and in the third group, the implant was left *in situ* throughout life. Parallel controls of pituitary intrarenal implants for different periods were also set up.

As seen in Table II, a similar incidence of mammary tumours (57 per cent) occurred whether mammary gland stimulation was maintained throughout life as compared with the limited period of the concurrent action with the carcinogen (46 per cent). Both 6 months and life-long stimulations did not affect the average latent period of tumour development.

Continuous mammary gland stimulation throughout life without additional carcinogen treatment induced mammary tumours in 13·6 per cent of $LAF_1$ mice carrying the intrarenal pituitary isograft and shortening of the period of extra mammary gland stimulation reduced this tumour incidence.

TABLE II.—*Maximum Threshold for Mammary Gland Stimulation in Mammary Tumorigenesis in $LAF_1$ Mice*

| Treatment | Mice bearing tumours/survivors | | Average latent period (days) |
|---|---|---|---|
| Pituitary graft continuously + MC × 6 | 40/70 | 57% | 370 |
| Pituitary graft 6 months + MC × 6 | 38/86 | 44% | 345 |
| Pituitary graft 3 months + MC × 6 | 34/75 | 45·6% | 350 |
| Pituitary graft continuously | 6/44 | 13·6% | 420 |
| Pituitary graft 6 months | 4/50 | 8% | 435 |
| Pituitary graft 3 months | 2/60 | 3% | 520 |
| MC × 6 | 1/45 | 2% | 375 |

MC = Methylcholanthrene.

*The effect of ovariectomy and adrenalectomy on the development of the preneoplastic lesions*

Preneoplastic lesions were induced in $LAF_1$ and DBA/2 mice by the concurrent action of mammary gland stimulation by intrarenal pituitary isograft and methylcholanthrene treatment for 3 months. The mice were then divided into three groups: In group 1, the pituitary implant was removed 10 days after the last MC application; in group 2, the implant and both ovaries were removed at the same time; and in group 3, a triple operation was performed involving the removal of the pituitary implant, both ovaries and both adrenals.

TABLE III.—*The Effect of Ovariectomy and Adrenalectomy on Further Growth of Preneoplastic Nodules in $LAF_1$ and DBA/2 Mice*

| | DBA/2 mice | | | $LAF_1$ mice | | |
|---|---|---|---|---|---|---|
| Treatment | Mice bearing tumours/survivors | | Average latent period (days) | Mice bearing tumours/survivors | | Average latent period (days) |
| Pituitary graft 15 weeks + MC × 6 | 39/50 | 78% | 210 | 35/74 | 47% | 350 |
| Pituitary graft 15 weeks + MC × 6 → ovex + adrex | 17/30 | 57% | 226 | 3/51 | 6% | 324 |
| Pituitary graft 15 weeks + MC × 6 → ovex | 14/26 | 54% | 210 | 3/47 | 6% | 417 |

Mc = Methylcholanthrene.
Ovex = Ovariectomy.
Adrex = Adrenalectomy.

As seen in Table III and Fig. 1, the removal of the ovaries and adrenals or of the ovaries alone inhibited mammary tumour development in $LAF_1$ mice—a 6 per cent incidence of mammary tumours in castrated mice or double operated animals, compared to 47 per cent in the intact treated mice. Contrary to $LAF_1$ mice, in DBA/2 mice only a slight decrease in mammary tumour development was observed—57 per cent in the operated mice, compared to 78 per cent in the intact mice. Similar effects on the further development of the preneoplastic induced lesions in $LAF_1$ and DBA/2 mice were obtained whether the mice were ovariectomized and adrenalectomized or only ovariectomized. The latent period of tumour development in DBA/2 mice was significantly shorter than in $LAF_1$ mice (Fig. 1). These results indicate that the concurrent treatment of mammary gland stimulation and carcinogen induce autonomous preneoplastic lesions in

DBA/2 mice, whereas the preneoplastic lesions in $LAF_1$ mice are dependent on the function of the ovaries for their further neoplastic transformation.

*The persistence of the preneoplastic lesions*

Preneoplastic lesions were induced in $LAF_1$ mice, after which the animals were ovariectomized. A third of these mice were regrafted 12 weeks later with one ovary beneath the right kidney capsule. (The functioning of the implant was checked by studying the vaginal smears of these mice. This revealed again the typical oestrus cycle for this strain, whereas the control ovariectomized mice showed a continuous dioestrus cycle.) As seen in Table IV, ovariectomy in-

FIG. 1.—Histogram of mammary tumour incidence. Each square represents a tumour.
Ovex = Ovariectomy.
Adrex = Adrenalectomy.

hibited mammary tumour development from the induced preneoplastic lesions. The reimplantation of one ovary affected these initiated preneoplastic dependent lesions—20 per cent of these mice developed mammary tumours, compared to a 4·6 per cent tumour incidence in ovariectomized hosts carrying such preneoplastic lesions (Table IV and Fig. 1).

TABLE IV.—*Resumption of Growth of the Preneoplastic Dependent Lesions in $LAF_1$ Mice*

| Treatment | Mammary tumour incidence: No. tumours/total | Mammary tumour incidence: Per cent | Average latent period (days) |
|---|---|---|---|
| Pituitary graft 15 weeks + MC × 6 | 35/74 | 47 | 350 |
| Pituitary graft 15 weeks + MC × 6 → ovariectomy | 2/43 | 4·6 | 440 |
| Pituitary graft 15 weeks + MC × 6 → ovex 12 weeks → ovary implant | 7/35 | 20 | 385 |
| Pituitary graft 15 weeks + MC × 6 → ovex 12 weeks → MtT graft | 0/30 | — | — |

MC = Methylcholanthrene.
Ovex = Ovariectomy.
MtT = Mammotropic pituitary tumour.

Another group of ovariectomized mice, in which preneoplastic lesions were induced, was grafted after a 3-month interval with a functioning mammotropic pituitary tumour secreting prolactin and growth hormone (Furth, Gadsden, Clifton and Anderson, 1956). This treatment failed to enhance the growth of the preneoplastic lesions ; no mammary tumours developed in this group (Table IV).

### DISCUSSION

A certain degree of mammary gland stimulation is known to be essential for mammary tumour development, with oestrogens playing a major role in stimulating mammary gland proliferation through its influence on the pituitary mammotropes. The primary difference between mouse strains exhibiting a low and high mammary tumour incidence may lie in the available hormones, especially oestrogens, capable of causing stimulation and nodule formation in the mammary tissue. The proliferating mammary gland cells are a more favourable substrate for methylcholanthrene penetration (Dao, Bock and Crouch, 1959). Comparing the vaginal smears of $LAF_1$ and DBA/2 mice, the more regular oestrus cycle of DBA/2 mice would suggest a normal level of oestrogen secretion, stimulating the mammary glands, which would allow mammary tumours to arise after methylcholanthrene application, whereas the $LAF_1$ mice, with the irregular oestrus cycle, could explain the refractoriness of the poorly stimulating mammary tissue (lacking the nodules) to methylcholanthrene treatment.

In the present experiment, once the preneoplastic transformation was induced, the continuation or discontinuation of mammary gland stimulation failed to change significantly the tumour incidence or its latency. Mammary gland stimulation seemed therefore of importance mainly in the initiation of mammary tumour induction in mice, rather than in promoting tumour development. Continuous mammary gland stimulation by subcutaneous isografts of hypophyses has been shown to lead to mammary tumour development (Loeb and Kirtz, 1939; Mühlbock and Boot, 1959). In the present experiments similar observations were obtained in mice carrying the intrarenal hypophysis implant throughout life or for a limited period of 6 months.

The induced preneoplastic lesions were found to be autonomous lesions in DBA/2 mice, progressing to malignant autonomous tumours in ovariectomized and adrenalectomized mice, but dependent on ovarian hormones for further development to autonomous mammary tumours in $LAF_1$ mice. The shorter latent period of mammary tumour development in DBA/2 mice also indicated the more favourable "inherited" hormonal influence (Bittner, 1942) in this strain, in which the preneoplastic transformation towards autonomy is completed within the 3 months of the concurrent treatment of mammary gland stimulation and methylcholanthrene application. Consequently, less time is required for full palpable tumour development in this case.

Similar results on the further development of the preneoplastic lesions in $LAF_1$ and DBA/2 mice were obtained when mice were ovariectomized and adrenalectomized or only ovariectomized, suggesting that the ovarian hormones are the main contributors to the autonomous neoplastic transformation: DBA/2 mice with a probable higher level of oestrogen secretion (reflected in the regular oestrus cycle) could enhance the neoplastic autonomous transformation of the preneoplastic lesions before the removal of the ovaries, whereas $LAF_1$ mice, with lower oestrogen levels (expressed by the irregular oestrus cycle) needed additional ovarian stimulation to complete this neoplastic autonomous transformation.

While the preneoplastic lesions induced in $LAF_1$ mice did not proceed to malignancy in the absence of the ovaries, regrafting of one ovary 3 months later, encouraged the persistence of the preneoplastic changes in the tissue, permitting some of them to proceed to palpable tumour growths. Andervont and Dunn (1964) have shown that preneoplastic changes, induced by diethylstilboestrol, could be further stimulated to neoplastic transformation after a longer interval of removal of the hormonal promoting factor than in our present experiment: A second exogenous hormone treatment after a 26-week interval was still effective in promoting the latent preneoplastic lesions. The induced preneoplastic lesions are, in certain strains, dependent lesions, requiring further hormonal stimulation to produce the autonomous variant. They can, in other words, remain in a "dormant" state when this additional hormonal stimulation is interrupted, and can be reactivated when additional hormonal stimulation is provided.

Furth and Kim (1961) have shown that in rats, a mammotropic pituitary tumour (MtT), secreting prolactin and growth hormone, when grafted into rats whose primary mammary tumour had regressed as a consequence of ovariectomy or hypophysectomy, caused resumption of progressive tumour growth. Implantation of MtT was therefore used in our experiment, as a parallel to the ovarian graft, in trying to enhance the development of dependent preneoplastic lesions towards autonomy. But MtT, unlike ovarian graft, proved ineffective in causing this transformation. These results might point to the possibility that ovarian oestrogens contribute to the development of mammary tumours, not only as stimulants to mammotrope secretion, but also as a direct effect on the autonomous neoplastic transformation in mice. However, more evidence for this concept is required.

### SUMMARY

The induction of preneoplastic mammary nodules and their hormonal requirements for progression to neoplasia were studied in the $LAF_1$ and DBA/2 strains of mice, which normally have a 0–3·5 per cent incidence of spontaneous mammary tumours, and which are considered to be free of the mammary tumour agent. $LAF_1$ mice were refractory to methylcholanthrene mammary carcinogenesis, whereas DBA/2 mice were susceptible—reflecting a different degree of primary mammary gland substrate, associated with their respective inherited hormonal patterns. This was checked by comparing the oestrus cycles in the two strains. Concurrent stimulation of mammary gland, through excess of prolactin secretion brought about by a pituitary intrarenal isograft, and acting in conjunction with methylcholanthrene applications, was effective in inducing preneoplastic mammary lesions in both strains. The mammary gland stimulation was only needed for the initiation of mammary tumour development, not for its promotion. The preneoplastic lesions induced in $LAF_1$ mice were found to depend, for their further neoplastic transformation, on the ovarian hormones; similar lesions induced in DBA/2 mice already constituted autonomous transformations, after the initial inductive treatment, having the capacity of developing into palpable tumours in ovariectomized and adrenalectomized hosts. The growth of the dependent preneoplastic lesions in ovariectomized $LAF_1$ mice could be restored by regrafting an ovary into these hosts, while failing to respond to prolactin secretion brought about by a mammotropic pituitary tumour grafted subcutaneously.

The author is indebted to Dr. W. E. Poel for his interest and valuable assistance in taking care of some phases of this work while the author was abroad; also to Dr. I. Berenblum for his encouragement and advice, and to Mr. S. Yecheskel for his competent technical assistance.

### REFERENCES

Andervont, H. B. and Dunn, T. B.—(1964) *J. natn. Cancer Inst.*, **33**, 143.
Bern, H. A. and Nandi, S.—(1961) *Prog. exp. Tumor Res.*, **2**, 99.
Bittner, J. J.—(1942) *Cancer Res.*, **2**, 710.
Dao, T. L., Bock, F. G. and Crouch, S.—(1959) *Proc. Soc. exp. Biol. Med.*, **102**, 635.
Desclin, L.—(1950) *Annls Endocr.*, **11**, 656.
Everett, J. W.—(1954) *Endocrinology*, **54**, 685.
Furth, J., Gadsden, E. L., Clifton, K. H. and Anderson, E.—(1956) *Cancer Res.*, **16**, 600.
Furth, J. and Kim, U.—(1961) in 'Biological Foundation of Cancer Control by Hormones'. New York (Academic Press), p. 259.
Haran-Ghera, N.—(1961) *Cancer Res.*, **21**, 790.—(1963) *Acta Un. int. Cancr.*, **19**, 765.
Loeb, L. and Kirtz, M. M.—(1939) *Am. J. Cancer*, **36**, 56.
Lyons, W. R., Li, C. H. and Johnson, R. E.—(1958) *Recent Prog. Horm. Res.*, **14**, 219.
Mühlbock, O. and Boot, L. M.—(1959) *Cancer Res.*, **19**, 402.